# A cost-effectiveness analysis of a community based CVD program in Sweden based on a retrospective register cohort

**DOI:** 10.1186/s12889-018-5339-3

**Published:** 2018-04-04

**Authors:** Lars Lindholm, Anna Stenling, Margareta Norberg, Hans Stenlund, Lars Weinehall

**Affiliations:** Department of Public Health and Clinical Medicine, Unit of Epidemiology and Global Health, Umeå University, Umeå, Sweden

**Keywords:** Prevention, Community-based, Cost-effectiveness

## Abstract

**Background:**

Several large scale community-based cardiovascular disease prevention programs were initiated in the 80s, and one was the Västerbotten Intervention Programme, Sweden. As an initial step in 1985, a pilot study was introduced in the Norsjö municipality that combined individual disease prevention efforts among the middle-aged population with community-oriented health promotion activities. All citizens at 30, 40, 50, and 60 years of age were invited to a physical examination combined with a healthy dialogue at the local primary health care centre. Västerbotten Intervention Program is still running following the same lines and is now a part of the ordinary public health in the county.

The purpose of this study is to estimate the costs of running Västerbotten Intervention Programme from 1990 to 2006, versus the health gains and savings reasonably attributable to the program during the same time period.

**Methods:**

A previous study estimated the number of prevented deaths during the period 1990–2006 which can be attributed to the programme. We used this estimate and calculated the number of QALYs gained, as well as savings in resources due to prevented non-fatal cases during the time period 1990 to 2006. Costs for the programmes were based on previously published scientific articles as well as current cost data from the county council, who is responsible for the programme.

**Result:**

The cost per QALY gained from a societal perspective is SEK 650 (Euro 68). From a health care sector perspective, the savings attributable to the VIP exceeded its costs.

**Conclusion:**

Our analysis shows that Västerbotten Intervention Programme is extremely cost-effective in relation to the Swedish threshold value (SEK 500000 per QALY gained or Euro 53,000 per QALY gained). Other research has also shown a favorable effect of Västerbotten Intervention Programme on population health and the health gap. We therefore argue that all health care organizations, acting in settings reasonably similar to Sweden, have good incentive to implement programs like Västerbotten Intervention Programme.

## Background

Several large scale community based cardiovascular disease (CVD) prevention programs were initiated in the late 80s including Minnesota and Pawtucket in US, North Karelia in Finland, Heartbeat Wales in UK and the Västerbotten Intervention Program (VIP) in Sweden. These programs have generated extensive and rich research, and VIP alone has been the source for hundreds of scientific articles. However, there is a striking absence of health economic perspectives in these publications. We have searched PubMed using different combinations of terms such as “cost-effectiveness” and “community based CVD prevention”, and found only six publications [1–6], (all from 1997 or earlier). One report that was based on the VIP [6] used intermediate measures, such as changes in cholesterol and blood-pressure, to predict eight-year cumulative incidence of coronary heart disease using a Framingham risk equation. From a societal perspective, the results ranged between savings and a cost of 1950 £ per year of life gained.

In general, a cost-effectiveness analysis requires endpoint data such as changes in morbidity and/or mortality, and these data can either be collected directly or modelled based on intermediate outcomes, e.g. changes in blood pressure or smoking habits. Neither of the routes are, however, easily accessible.

Effectiveness assessment based on impact on morbidity and mortality presuppose a sustainable intervention, a structure in place which enables long-term follow up, and a sufficiently large scale to be able to measure impact on rare events such as mortality. These prerequisites require a patient funder who is willing to support an intervention for a decade or more prior to seeing any tangible returns. Against this background, the concept of “modeling for decision-making” has become popular. This involves predicting final outcomes on the basis of intermediate ones by applying the philosophy of evidence based medicine; to use the best available data when you are forced to make a decision. Among the advantages of this methodology are the relatively low cost and timeliness. Decision-makers therefore receive a relatively prompt answer, and do not risk funding an intervention for a decade that in the end turns out to be worthless. Modelling also has its disadvantages, and Burgers et al. [7] have recently completed a review of the traps encountered when creating models in the field of CVD. In summary, they argue that many models lack validity because appropriate data are not available.

Fortunately, VIP is one of the few community-based CVD programs that meet all the criteria discussed above for making an economic analysis based on end-points feasible. Such a publication would fill two gaps in the literature: a) the absence of studies from the last 20 years and b) the absence of studies based on empirically confirmed mortality.

### Aim of the study

To estimate the costs of running VIP from 1990 to 2006, versus the health gains and savings reasonably attributable to the program during the same time period.

### Setting - Västerbotten intervention programme

Coronary heart disease mortality started to gradually increase in Sweden starting in the early 1900s. This trend occurred more slowly in northern Sweden and was not noted until the 1940s. Epidemiological studies from the 1970s showed that Västerbotten County had the highest age-adjusted CVD mortality rate in Sweden (ages 15 to 74) and that this rate was 40% higher than the county with the lowest mortality.

In response to these alarming epidemiological reports, in 1984 the Västerbotten County Council decided to develop a model for a long term population-oriented program for the prevention of both cardiovascular disease and diabetes. The program was based on integrated cooperation within local communities with primary health care as the coordinating hub.

As an initial step in 1985, a pilot study was introduced in the Norsjö municipality that combined individual disease prevention efforts among the middle-aged population with community-oriented health promotion activities. All citizens at 30, 40, 50, and 60 years of age were invited to a physical examination combined with a healthy dialogue at the local primary health care centre. A 10-year evaluation showed that the age- and education-adjusted CVD mortality risk, based on the North Karelia CHD risk equation, was reduced by 36% in Norsjö, compared to a 1% reduction in the reference area (Northern Sweden MONICA Study). This risk reduction was found to have occurred primarily in the low education group [8].

Based on experience from the pilot study, the core intervention concept ─ physical examination, survey, and health dialogue ─ was implemented in all the county’s municipalities during the early 1990s. This collective effort became known as the Västerbotten Intervention Programme (VIP). A detailed description of the design of the VIP, CVD risk factor measurements and questionnaire data, support of primary care providers, and development according to medical evidence, has been published previously. VIP was included in the regular Västerbotten primary care mission from 1995 [9].

During the 17 years (1990–2006) reported in this study, the participation rate gradually increased from about 55 to 65%, with only small socio-economic differences between participants and non-participants [10].

### The VIP-intervention: Individual health dialogue

Nurses provided feed-back to participants based on the results of CVD risk factor measurements, health, lifestyle habits, and socioeconomic and psychosocial factors. Initially, this occurred in a unidirectional fashion with the nurse lecturing the participant. Gradually, however, this communication was developed into a dialogue, based on the concept of motivational interviewing. The participant’s risk profile was visualized in the shape of a star, where greater risk was indicated by blunt tips and a low risk was shown as a star with sharp tips. This pedagogical tool is aimed at facilitating the understanding of relationships between life style habits and CVD risk factors. The goal was to motivate the participants with a low risk factor burden to maintain healthy habits, and to support and motivate those with multiple risk factors to modify their behaviour. If appropriate, follow-up visits or referral to the family physician (usually a GP) for further evaluation and treatment was recommended.

### Risk factor monitoring

According to published reports, from 1990 to 2007 smoking prevalence among VIP participants significantly decreased both for men and women [11]. The general trend of increasing obesity has slowed, the prevalence of hypercholesterolemia [12] and hypertension [13] have significantly decreased, and the level of physical activity has increased [14]. Over half of the participants with poor self-reported health at base-line have, at the 10-year follow up, reported improved self-reported health [15]. However, at the 10-year follow up, the average level of fasting glucose has increased by 0.5 mmol/L, which is also reflected in an increase in diabetes prevalence, especially among men [16]. Among VIP participants, there is a significant difference in risk factor burden between educational levels.

## Methods

**Fig. 1:**
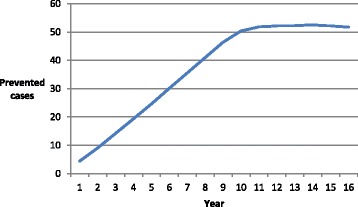
Estimated numbers of prevented deaths for each year in the period under study

Blomstedt et al. [17] estimated both total and CVD mortality impact from two perspectives; target group and participants, with the target group (intention to treat) treated as the main analysis. This same convention is used in our current analyses. They estimated the total mortality gains for the period 1990–2006 to be 587 prevented premature deaths, out of which 109 were CVD. We have allocated the total amount of prevented deaths to years in proportion to the number of person-years recorded in each calendar year (Fig. 1).

### Life years gained

Life years gained is determined as the time elapsed between July 1 (mid-year) the year the death was prevented and December 31, 2006. Prevented deaths 2006 thus gain 0.5 years, 2005 gain 1.5 years etc.

### Quality adjusted life years gained

Life-years is transformed to quality-adjusted life years (QALYs) via multiplication by a QALY-weight determined in a population survey in Stockholm, Sweden between 1998 and 2002 [18]. This is the “health level” the average Swede can expect conditional on age and sex. For both sexes, the QALY-weight is quite stable from middle age to 74, with a tendency for a slight increase in the years after retirement. Males usually report a higher level than females, and in our calculations we have used 0.82 for males and 0.78 for females.

### QALYs gained and savings due to the prevention of non-fatal CVD events

Prevented premature CVD deaths have been split into CHD and stroke because the loss of QALY’s and the treatment costs are different for non-fatal cases of CHD and stroke respectively (Table 1). A reasonable rule of thumb for the whole period is 2/3 CHD and 1/3 of stroke.

**Table 1:** Assumptions regarding case fatality rate, loss of QALYs per case and cost per case

	Proportion	Fatal cases	Case fatality rate	Non fatal cases	Loss of QALY per case	Cost per case
CVD	1					
All		109				
Male		58				
Female		51				
CHD	2/3		20/100	360		
All		72			0.051	
Male		39				SEK 250000 (m)
Female		33				SEK 180000 (f)
Stroke	1/3		18/100	206		First time event:
All		37			0.145	
Male		19				SEK 787000 (m)
Female		18				SEK 695000 (f)

To estimate the number of non-fatal cases for CHD we used the fatality case rate/lethality in 28 day [19], and did the same for stroke [20]. The proportion of “first-time” incidence cases was used (=70%) for stroke because the most recent cost-estimates are based on this fraction [19].

Lindgren et al. [21] have measured the loss of QALYs in the years following the event, and report 0.145 for stroke and 0.051 for acute coronary syndrome.

Ghatnekar and Carlsson [22] have measured the societal costs following a first time ever stroke using a life-time approach and societal perspective. The 2015 values inflated by the consumer price index for males and females are SEK 787000 and SEK 695000 respectively (Euro 1 = SEK 9,5 and USD 1 = SEK 8).

Määtää [23] have studied the health care costs associated with myocardial infarction and estimated the cost per event to be SEK 250000 for males and SEK 180000 for females.

### Intervention costs

Costs for VIP are shown in Table 2. A detailed cost analysis was published in 1994 [24] and the cost to the health care center was estimated to be SEK 720 per patient. The components of this estimate included time required by the general practitioner, nurse and assisting nurse, plus laboratory costs. A similar analysis completed for 2015 showed that the cost had dropped to SEK 436. The main reasons for this decline in costs are changes in the VIP protocol, with the health counseling being done by a nurse, and decreased total visit time attributable to new laboratory test methods.

**Table 2:** Costs per participant (SEK, year 2015 values) for the intervention in 1992 and 2015

Component	1992^a^	2015	Rounded “average” 1990–2006
Cost at HCC per participant	720	436	600
Total program overhead costs for management, analysis etc.		1 363,600	210
Program costs per participant		210	100
Time costs per participant	620	1575	1000
Total costs	1340	2221	1700

^a^Costs have been inflated by the consumer price index

The second item in 2015 is resources for coordination, analysis and education. The total cost is SEK 1.4 million and the cost per participant is equal to 210 SEK. Corresponding information from 1994 is not available.

Finally, time costs are estimated for the 2 years. In 2015, it was assumed that the first visit took 2 hours, and travel required another hour. Further, we assumed that 75% of participants made a second visit, requiring another hour plus one travel hour. Using the average Swedish hourly labour cost in 2015 – SEK 350 [25], the total time costs for the two visits are estimated to be 1575 SEK. Up to 2009, only one visit was standard and the time cost in 1992 was estimated to be 445 SEK.

In our retrospective analysis (Table 2), we have used “average” values for the period, i.e. weighting the two estimates together according to their representativeness for the period under study (Table 2).

All costs are expressed at the price level in 2015, i.e. costs from previous years are inflated by the consumer price index to reflect year 2015 values.

## Result

The total intervention costs for VIP for the health care sector are SEK 67,4 million (96,306 examinations times SEK 700) and SEK 192,6 (96,306 examinations times SEK 2000) from a societal perspective. QALYs gained between 1990 and 2006 are estimated to be 2904, with 2856 attributable to reduced mortality (Table 3).

**Table 3:** Estimated health gains and savings due to VIP during the period 1990 to 2006

	Change in total mortality	Prevented Non-fatal CVD	
Cases prevented		Stroke	CHD
Men	301	106	195
Women	286	100	165
All	587	206	360
Life years gained			
Men	1834		
Women	1733		
All	3567		
QALYs gained		Stroke	CHD
Men	1504		
Women	1352		
All	2856	29,9	18,4
Savings:		Stroke	CHD
a. Health care			
Male		15,5	49,1
Female		13,7	29,9
b. Municipalities			
Male		19,4	
Female		24,2	
c. Production gains			
Male		26,1	
Female		12,3	
d. Total		111,2	78,9
Male		61	49,1
Female		50,2	29,9

From a societal perspective the savings amount to SEK 190,1 million and from a health care perspective they amount to SEK 108,2 million. Thus, from a health care sector perspective, the savings attributable to the VIP exceeded its costs, while the cost per QALY gained from a societal perspective is SEK 650.

## Discussion

The main finding is that VIP is an extremely cost-effective intervention. From a health care perspective, savings were about 50% larger than costs during the period under study. From a societal perspective, costs and savings were the same.

### Methodological considerations

The approach used in this study has as a notable strength the use of retrospective data of good quality. However, our approach has also the following limitations:

*While it was possible to estimate the number of non-fatal cases of CVD, it was not possible to estimate the number of non-fatal cases of other diseases. The fact that non-fatal cases of other diseases were prevented as well, but were not accounted for in our analysis, strongly implies a significant underestimation of the health gained and “savings” in the period under study.
*Our analysis was truncated 2006, which also results in an underestimation of health gains. In the end of the period (2006) 587 more individuals were alive than expected, and these continues to live beyond 2006.
*Conversely, the absence of a life-long perspective also gives a misleading picture of “savings”. The prevention of premature deaths and lived years beyond the time window of this study means that costs for treating the diseases which finally cause the deaths have not been taken into consideration. It is well-known that a significant portion of health care resources consumed during a lifetime arise in the last year prior to death. When a potential premature death due to CVD is avoided, a competing cause of disease and death in old-old age, eg. years of dementia followed by a death caused by cancer, must inevitably take its place. The resulting peak in health care costs are therefore postponed beyond our study window, and these later health care costs could perhaps be of the same magnitude as the savings we have accounted for. The distance in time to “old-old” age makes it necessary to discount these later costs, which will lower the present value. There is, in general, a gain connected to postponing health care costs into the future. Among the reasons for this are that future health care will likely have access to larger resources than health care today, and also the possibility that more cost-effective treatment methods have been developed in the meantime.
*Retrospective costing is unusual in cost-effectiveness analyses, and its use poses some methodological challenges. The beginning of the study period (1990) was more than 25 years ago, and therefore poses a challenge to stakeholders’ memories. The study period ends more than 10 years ago, and data collected today is probably not completely valid for the end of our period under study. We must therefore rely heavily on studies published in 1994 and 1996 [6, 24], to provide estimates of the cost structure that was present at the beginning of the VIP. However, VIP has changed gradually, and some local variations in the protocol have been allowed and even encouraged. For these reasons, also the recent cost estimate from 2015 has been used despite it refers to VIP 9 years after the end of the studied period. We think our “weighted average” captures some of the dynamics inherent in VIP. The used “average” makes the cost higher than the estimates published in 1994, so there is a kind of sensitivity analysis built in.

In sum, the limitations in our approach in comparison with “modelling” have certainly underestimated the health gains. Savings during the period of study are underestimated while costs beyond the period are not taken into account. It is not possible to calculate the net effect of these two limitations. However, modelling can’t be considered “the gold standard” simply because it is the most common approach. As summarized in the background, modelling is a technique with its own pro and cons.

### Is VIP’s cost-effectiveness unique?

Any comparison must be limited to intervention programs that are very similar to VIP, following the principles for health care technology assessment. One such study is the CVD prevention program in Franklin County, Maine US [26]. In a retrospective evaluation for the period 1970–2010, this program was found to have improved control of hypertension and elevated cholesterol, and also improved smoking quit rates. These changes in risk-factors contributed to an observed decrease in mortality rates and hospital utilization in the study area. Unfortunately, the costs for this intervention are not reported.

The Ebeltoft Health Promotion Project reported data on life expectancy and costs [26, 27]. In summary, they used a kind of cost-minimization analysis that showed that the intervention increased life expectancy without increasing costs.

The cost-effectiveness of the British Oxcheck and British family heart studies have also been evaluated [28]. The calculation is a bit speculative and estimates the change in life expectancy due to improved risk factors. The results depend heavily on the assumed time period for duration of effect. The cost per life year gained for men in the British family heart study ranged between £ 900 (20 years duration) to £ 24,400 (1 year). Corresponding values for Oxcheck were £ 900 and £ 20,900 respectively.

### Policy consequences

A CVD prevention program that is very similar to VIP has been running in the South of Sweden. This is the “Live for Life” program in the Counties of Skaraborg and Jönköping. The effects of this intervention on CVD risk factors and disease outcomes, which are similar to those from VIP, have been reported, but there have as yet been no health economic evaluations [29–31].

There has been a long-running discussion in the scientific community about the possibility of using prevention programs to reduce the CVD burden in the population. The conclusion based on, for example, Cochrane evaluations of multiple RCTs, is that CVD prevention efforts have not been effective [32, 33].

However, these evaluations do not include the type of intervention that VIP represents and are therefore not relevant here. VIP utilizes both individual-oriented and population-oriented strategies. Primary care’s annual invitations to all citizens age 40, 50 and 60 years to a CVD-focused health survey and personal health dialogue interact with a strong public health interest and preparedness for behavioural modification.

The VIP model is integrated within the existing primary care delivery structure, anchored in the local community with support from both political decision-makers and the general population, and has lasted for more than 25 years. It is like the Swedish child health program in that it is part of the basic health care system.

Our assessment is that the outcomes in our previous reports based on VIP [6, 8, 17] are a well-founded basis for policy development and for decisions regarding implementation of interventions similar to VIP. In light of the health economic outcomes presented by this study, our conclusion is that this model is capable of influencing the population’s risk factor burden and mortality risk in a cost-effective manner.

## Conclusions

hese analyses, in conjunction with a previously published report [6], show that VIP is extremely cost-effective in relation to the Swedish threshold value (SEK 500000 per QALY gained). Other research [8, 17] has also shown a favorable effect of VIP on population health and the health gap. We therefore argue that all health care organizations, acting in settings reasonably similar to Sweden, have a good incentive to implement programs like VIP.

## Abbreviations

CVD: Cardiovascular disease
QALY: Quality Adjusted Life Years
VIP: Västerbotten Intervention Programme

## References

[1] Altman DG, Flora JA, Fortmann SP, Farquhar JW (1987). The cost-effectiveness of three smoking cessation programs. Am J Public Health.

[2] Farquhar JW, Fortmann SP, Flora JA (1990). Effects of community-wide education on cardiovascular disease risk factors. The Stanford Five-City project. JAMA.

[3] Nissinen A, Tuomilehto J, Kottke TE, Puska P (1986). Cost-effectiveness of the north Karlia hypertension program 1972-1977. Med Care.

[4] Tosteson ANA (1997). Cost-effectiveness of population-wide educational approaches to reduce serum cholesterol levels. Circulation.

[5] Phillips CJ, Prowle MJ (1993). Economics of a reduction in smoking: case study from heartbeat Wales. J Epidemiol Community Health.

[6] Lindholm L, Rosén M, Weinehall L, Asplund K (1996). Cost-effectiveness and Equity of a community based cardiovascular disease prevention programme in Norsjö, Sweden. J Epidemiol Community Health.

[7] Burgers LT, Redekop WK, Severens JL (2014). Challenges in Modelling the cost effectiveness of various interventions for cardiovascular disease. PharmaEconomics.

[8] Weinehall L, Hellsten G, Boman K, Hallmans G, Asplund K, Wall S (2001). Can a sustainable community intervention reduce the health gap? 10-year evaluation of a Swedish community intervention program for the prevention of cardiovascular disease. Scand J Public Health Suppl.

[9] Norberg M, Wall S, Boman K, Weinehall L. The Vasterbotten intervention Programme: background, design and implications. Glob Health Action. 2010;3 10.3402/gha.v3i0.4643. 10.3402/gha.v3i0.4643 PMC2844807 20339479

[10] Norberg M, Blomstedt Y, Lonnberg G, Nystrom L, Stenlund H, Wall S (2012). Community participation and sustainability--evidence over 25 years in the Vasterbotten intervention Programme. Glob Health Action.

[11] Norberg M, Lundqvist G, Nilsson M, Gilljam H, Weinehall L. Changing patterns of tobacco use in a middle-aged population - the role of snus, gender, age, and education. Glob Health Action. 2011;4 10.3402/gha.v4i0.5613 PMC3118776 21695071

[12] Ng N, Johnson O, Lindahl B, Norberg M. A reversal of decreasing trends in population cholesterol levels in Västerbotten County, Sweden. Glob Health Action. 2012;5 10.3402/gha.v5i0.10367 PMC3313585 22468143

[13] Ng N, Carlberg B, Weinehall L, Norberg M. Trends of blood pressure levels and management in Västerbotten County, Sweden, during 1990-2010. Glob Health Action. 2012;5 10.3402/gha.v5i0.18195 PMC3409341 22855645

[14] Ng N, Soderman K, Norberg M, Ohman A. Increasing physical activity, but persisting social gaps among middle-aged people: trends in northern Sweden from 1990 to 2007. Glob Health Action. 2011;4 10.3402/gha.v4i0.6347 PMC3144737 21799669

[15] Blomstedt Y, Emmelin M, Weinehall L (2011). What about healthy participants? The improvement and deterioration of self-reported health at a 10-year follow-up of the Vasterbotten intervention Programme. Glob Health Action.

[16] Lindahl B, Stenlund H, Norberg M. Increasing glucose concentrations and prevalence of diabetes mellitus in northern Sweden, 1990–2007. Glob Health Action. 2010;3 10.3402/gha.v3i0.5222 PMC2966476 21042431

[17] Blomstedt Y, Norberg M, Stenlund H, Nyström L, Lönnberg G, Boman K, Wall S, Weinehall L (2015). Impact of a combined community and primary care prevention strategy on all-cause and cardiovascular mortality: a cohort analysis based on 1 million person-years of follow-up in Västerbotten County, Sweden, during 1990-2006. BMJ Open.

[18] Burström K, Johannesson M, Rehnberg C (2007). Deteriorating health status in Stockholm 1998-2002: results from repeated surveys using the EQ5D. Qual Life Res.

[19] Myocardial infarctions in Sweden 1994–2014. The Board for Health and Welfare 2015.

[20] Stegmayr B and Asplund K. Ökad överlevnad vid stroke men oförändrad risk att insjukna. Läkartidningen nr 44, volym 100, 3492–3498, 2003. 14651007

[21] Lindgren P, Kahan T, Poulter N, Buxton M, Svarvar P, Dahlöf B, Jönsson B, on behalf of the ASCOT investigators (2007). Utility loss and indirect costs following cardiovascular events in hypertensive patients: the ASCOT health economic substudy. Eur J Health Econ.

[22] Ghatnekar O and Steen Carlsson K. Costs for getting a stroke in 2009. An incidence-based study. IHE report 2012:2.

[23] Määttä S, Schenk-Gustafsson K, Trollvik M, Karlsson Å, Evengård B (2015). Males have higher cost when getting myocardial infarction and appendicit. Lakartidningen.

[24] Lindholm L, Rosén M, Hellsten G (1994). Are people willing to pay for a community-based preventive program? - a case study from Norsjö, Sweden. Int J Technol Assess Health Care.

[25] Record NB (2015). Community-Wide Cardiovascular Disease Prevention Programs and Health Outcomes in a Rural County, 1970–2010. JAMA.

[26] Rasmussen SR (2007). Preventive health screenings and health consultations in primary care increase life expectancy without increasing costs. Scand J Pub Health.

[27] Lauritzen T (2008). Health tests and health consultations reduced cardiovascular risk without psychological strain, increased healthcare utilization or increased costs. Scand J Pub Health.

[28] Wonderling D, Langham S, Buxton M, Normand C, McDermott C (1996). What can be concluded from the Oxcheck and British family heart studies: commentary on cost effectiveness analyses. BMJ.

[29] Persson LG, Lingfors H, Nilsson M (2015). Lifestyle, biological risk markers, morbidity and mortality in a cohort of men 33 - 42 years old at baseline, after 24-year follow-up of a primary health care intervention. Open J Prev Med.

[30] Lingfors H, Lindström K, Persson LG (2003). Lifestyle changes after a health dialogue. Results from the live for life health promotion programme. Scand J Prim Health Care.

[31] Lingfors H, Persson LG, Lindström K (2009). Effects of a global health and risk assessment tool for prevention of ischemic heart disease in an individual health dialogue compared with a community health strategy only - results from the Live for Life health promotion programme. Prev Med.

[32] Dyakova M, Shantikumar S, Colquitt JL, Drew CM, Sime M, MacIver J (2016). Systematic versus opportunistic risk assessment for the primary prevention of cardiovascular disease. Cochrane Database Syst Rev.

[33] Krogsboll LT, Jorgensen KJ, Gronhoj Larsen C, Gotzsche PC (2012). General health checks in adults for reducing morbidity and mortality from disease: Cochrane systematic review and meta-analysis. BMJ.

